# Metaplastic Regulation of CA1 Schaffer Collateral Pathway Plasticity by Hebbian MGluR1a-Mediated Plasticity at Excitatory Synapses onto Somatostatin-Expressing Interneurons^1^,^2^,^3^

**DOI:** 10.1523/ENEURO.0051-15.2015

**Published:** 2015-08-21

**Authors:** Cristina Vasuta, Julien Artinian, Isabel Laplante, Sarah Hébert-Seropian, Karim Elayoubi, Jean-Claude Lacaille

**Affiliations:** Groupe de Recherche sur le Système Nerveux Central and Department of Neuroscience, Faculty of Medicine, Université de Montréal, Montreal, Quebec H3T 1J4, Canada

**Keywords:** hippocampus, LTP, metaplasticity, mGluR1a, somatostatin-interneuron

## Abstract

Cortical GABAergic interneurons represent a highly diverse neuronal type that regulates neural network activity. In particular, interneurons in the hippocampal CA1 oriens/alveus (O/A-INs) area provide feedback dendritic inhibition to local pyramidal cells and express somatostatin (SOM). Under relevant afferent stimulation patterns, they undergo long-term potentiation (LTP) of their excitatory synaptic inputs through multiple induction and expression mechanisms. However, the cell-type specificity of these different forms of LTP and their specific contribution to the dynamic regulation of the CA1 network remain unclear. Here we recorded from SOM-expressing interneurons (SOM-INs) in the O/A region from SOM-Cre-Ai3 transgenic mice in whole-cell patch-clamp. Results indicate that, like in anatomically identified O/A-INs, theta-burst stimulation (TBS) induced a Hebbian form of LTP dependent on metabotropic glutamate receptor type 1a (mGluR1a) in SOM-INs, but not in parvalbumin-expressing interneurons, another mainly nonoverlapping interneuron subtype in CA1. In addition, we demonstrated using field recordings from transgenic mice expressing archaerhodopsin 3 selectively in SOM-INs, that a prior conditioning TBS in O/A, to induce mGluR1a-dependent LTP in SOM-INs, upregulated LTP in the Schaffer collateral pathway of pyramidal cells. This effect was prevented by light-induced hyperpolarization of SOM-INs during TBS, or by application of the mGluR1a antagonist LY367385, indicating a necessity for mGluR1a and SOM-INs activation. These results uncover that SOM-INs perform an activity-dependent metaplastic control on hippocampal CA1 microcircuits in a cell-specific fashion. Our findings provide new insights on the contribution of interneuron synaptic plasticity in the regulation of the hippocampal network activity and mnemonic processes.

## Significance Statement

Long-term potentiation (LTP) is an important cellular mechanism of learning and memory. Although it has been extensively characterized in principal cells of the hippocampus, it also occurs in inhibitory GABAergic interneurons, known to orchestrate hippocampal network activity. Interneurons are highly diverse and many subtypes are distinguished, endowed with distinct functions. However, their cell-type-specific contribution and how LTP in these interneurons regulate hippocampal CA1 microcircuits remain open questions. Here, we found that LTP occurring in the Schaffer collateral pathway of CA1 pyramidal cells was upregulated by prior induction of mGluR1a-dependent LTP in somatostatin-expressing interneurons. These results reveal a metaplastic control of the hippocampal CA1 network which can help to understand interneuron subtype-specific contribution in hippocampus-dependent learning and memory.

## Introduction

Information processing within the cerebral cortex relies on complex microcircuits of interconnected excitatory glutamatergic principal cells and inhibitory GABAergic interneurons. Comprising 10–20% of the total cortical neuronal population, GABAergic interneurons dynamically regulate neural networks by controlling the amount of excitation that neurons receive, by synchronizing their outputs and supporting brain oscillations (for review, see Mann and Paulsen, 2007; Klausberger and Somogyi, 2008). GABAergic interneurons are highly heterogeneous and multiple subtypes can be distinguished on the basis of their morphological, neurochemical, physiological, and developmental properties, which is particularly well documented in the hippocampus (Tricoire et al., 2011; Hosp et al., 2014; for review, see Freund and Buzsáki, 1996; Klausberger and Somogyi, 2008; Kepecs and Fishell, 2014).

Long-term potentiation (LTP) is an activity-dependent long-lasting change of the synaptic strength and is thought to be a key element in the cellular processes of learning and memory (for review, see Kandel et al., 2014; Lynch et al., 2014). Although LTP presents relatively stereotyped mechanisms in excitatory principal neurons, several different forms of LTP have been described at excitatory synapses onto hippocampal GABAergic interneurons (Ouardouz and Lacaille, 1995; Hainmuller et al., 2014; for review, see Pelletier and Lacaille, 2008; Kullmann et al., 2012; Topolnik, 2012). Particularly interesting are interneurons from the CA1 region, which have their cell body located in the stratum oriens, provide a dendritic inhibition to their pyramidal and interneuron targets and express somatostatin (SOM; also called SST; Tricoire et al., 2011; for review, see Freund and Buzsáki, 1996; Klausberger and Somogyi, 2008; Müller and Remy, 2014). Notably, NMDA receptor (NMDAR)-independent LTP, which depends on the type 1a metabotropic glutamate receptor (mGluR1a; Perez et al., 2001; Lapointe et al., 2004; Le Duigou and Kullmann, 2011) and on a postsynaptic Ca^2+^ rise from multiple sources (Topolnik et al., 2006; Lamsa et al., 2007; Oren et al., 2009; Croce et al., 2010), has been described in interneurons from the CA1 oriens/alveus region (O/A-IN) and in SOM-expressing interneurons (SOM-INs) from the O/A region (Szabo et al., 2012). Furthermore, LTP occurrence and rules appear to be highly cell-type-specific (Perez et al., 2001; Lamsa et al., 2007; Nissen et al., 2010; Szabo et al., 2012), adding an important level of complexity in our understanding of interneuron diversity.

At the network level, hippocampal CA1 O/A-INs are mainly activated by recurrent excitation from local pyramidal cells and act in an integrator mode as they inhibit distal dendrites in proportion to the rate of the synaptic inputs (Pouille and Scanziani, 2004). Furthermore, SOM-INs have been shown to specifically and dynamically regulate input-output transformations and to gate burst firing in pyramidal cells (Lovett-Barron et al., 2012). However, although oriens-lacunosum/moleculare (OLM) cells, a subset of SOM-INs in the O/A region, inhibit the pyramidal distal dendritic tuft, they also inhibit Schaffer collateral-associated GABAergic interneurons (Leão et al., 2012). Indeed, specific activation of OLM cells promotes a disinhibition of pyramidal neurons, increasing their responsiveness to Schaffer collateral inputs, and facilitates LTP at these synapses. In addition, these cells prevent LTP in the temporo-ammonic pathway, conferring them the ability to bidirectionally modulate CA1 plasticity (Leão et al., 2012). But how long-term plasticity occurring at excitatory synapses onto SOM-INs modulates network activity of pyramidal cells remains largely unknown. mGluR1a-dependent LTP occurring at excitatory inputs onto O/A-INs increases GABAergic inhibition of principal neurons (Lapointe et al., 2004) and NMDAR-dependent LTP induced in CA1 stratum radiatum interneurons maintains the temporal fidelity of input and output signals of pyramidal cells (Lamsa et al., 2005). Beyond the proper characterization of the different forms of LTP occurring in multiple interneuron subtypes, their specific contribution to the CA1 network regulation, especially in gating of LTP at Schaffer collateral-pyramidal cell synapses, remains an open question.

In this study, we address this question, first by assessing the cell-specificity of Hebbian mGluR1a-dependent LTP in two populations of interneurons within the hippocampal CA1 area: the SOM-INs and the parvalbumin-expressing interneurons (PV-INs), using mice lines expressing Cre recombinase under the control of either the SOM or the PV promoter. We found that theta-burst stimulation (TBS) induced Hebbian mGluR1a-dependent LTP in SOM-INs in a cell-specific fashion. Next, we establish the functional significance of the SOM-IN synaptic plasticity for network activity. We uncovered that prior induction of this form of LTP at excitatory synapses onto SOM-INs upregulated LTP in the Schaffer collateral pathway of CA1 pyramidal cells, establishing a cell-specific metaplastic control of the CA1 microcircuit by SOM-INs.

## Materials and Methods

All animal procedures and experiments were performed in accordance with the Université de Montréal animal care committee’s regulations.

### Transgenic mice lines

SOM-IRES-Cre mice were kindly provided by Z. J. Huang (Cold Spring Harbor Laboratory, Cold Spring Harbor, NY; JAX no. 013044; Taniguchi et al., 2011). PV-Cre mice (JAX no. 008069), the Cre-reporter expressing the enhanced yellow fluorescent protein (EYFP) Ai3 mice (JAX no. 007903) and the ArChR3/GFP Ai35 mice (JAX #012735) were purchased from Jackson Laboratories. Experiments were performed in mice of either sex.

### Interneuron distribution and immunofluorescence

To fluorescently label SOM-INs, heterozygous SOM-IRES-Cre;Ai3-EYFP mice were obtained by crossing SOM-IRES-Cre and Ai3-EYFP mice. To label PV-INs, heterozygous PV-Cre;Ai3-EYFP mice were generated by crossing PV-Cre and Ai3-EYFP mice. Distribution of EYFP-labeled interneurons and colocalization with somatostatin or parvalbumin were determined by combination of fluorescence microscopy and immunohistochemistry. SOM-IRES-Cre;Ai3-EYFP mice (3- to 5-weeks-old) and PV-Cre;Ai3-EYFP mice (6- to 8-weeks-old) were deeply anesthetized intraperitoneally with sodium pentobarbital (MTC Pharmaceuticals), perfused transcardially with ice-cold 0.1m phosphate buffer (PB) and 4% paraformaldehyde in 0.1m PB (PFA) and the brain isolated. Postfixed brains were cryoprotected in 30% sucrose and coronal brain sections (50 µm thick) were obtained using a freezing microtome (Leica SM200R). Sections were permeabilized with 0.2-0.5% Triton X-100 in PBS (15 min) and unspecific binding was blocked with 10% normal goat serum in 0.1-0.3% Triton X-100/PBS (1 h). Rabbit polyclonal somatostatin 28 (1/2000; Abcam) or mouse monoclonal parvalbumin (1/5000; Millipore) antibodies were incubated overnight at 4°C. Sections were subsequently incubated at room temperature with AlexaFluor 594-conjugated goat anti-rabbit IgGs (1/500; 90 min; Jackson ImmunoResearch Laboratories) or rhodamine-conjugated goat anti-mouse IgG1 (1/200; 90 min; Jackson ImmunoResearch Laboratories). Hippocampal sections were examined using a Nikon microscope (Nikon Eclipse E600) equipped with epifluorescence and images were acquired with the Simple PCI software (CImaging Systems).

### Slices and whole-cell recordings

Hippocampal slices were prepared from 4- to 8-week-old SOM-IRES-Cre;Ai3-EYFP mice and 6- to 8-week-old PV-Cre;Ai3-EYFP mice. Animals were anesthetized with isoflurane and the brain was rapidly excised and placed in ice-cold sucrose-based cutting solution saturated with 95% O_2_ and 5% CO_2_ containing the following (in mm): 87 NaCl, 2.5 KCl, 1.25 NaH_2_PO_4_, 7 MgSO_4_, 0.5 CaCl_2_, 25 NaHCO_3_, 25 glucose, 11.6 ascorbic acid, 3.1 pyruvic acid, and 75 sucrose, pH 7.4, and 295 mOsmol. A block of tissue containing the hippocampus was prepared and transverse hippocampal slices (300 μm thick) were cut on a vibratome (Leica VT1000S). Slices were transferred to oxygenated artificial CSF (ACSF) at room temperature containing the following (in mm): 124 NaCl, 2.5 KCl, 1.25 NaH_2_PO_4_, 4 MgSO_4_, 4 CaCl_2_, 26 NaHCO_3_, and 10 glucose, pH 7.3–7.4, and 295–305 mOsmol, allowed to recover for at least 1 h, and transferred for experiments to a submersion chamber perfused (2 ml/min) with ACSF at 31 ± 0.5°C. Prior to recordings, CA1 and CA3 regions were isolated by a surgical cut. EYFP-expressing CA1 interneurons were identified using an upright microscope (Nikon Eclipse, E600FN), equipped with a water-immersion long-working distance objective (40×, Nomarski Optics), epifluorescence and an infrared video camera. Whole-cell voltage-clamp recordings were obtained using borosilicate glass pipettes (3-6 MΩ) filled with intracellular solution containing the following (in mm): 130 CsMeSO_3_, 5 NaCl, 1 MgCl_2_, 10 phosphocreatine, 10 HEPES, 2 ATP-Tris, 0.4 GTP-Tris, 0.1 spermine, 2 QX314, and 0.1% biocytin, pH 7.2–7.3, and 275–285 mOsmol. For whole-cell current-clamp recordings of intrinsic properties, the intracellular solution contained the following (in mm): 150 K-gluconate, 3 MgCl_2_, 0.5 EGTA, 10 HEPES, 2 MgATP, 0.3 NaGTP, and 0.1% biocytin, pH 7.4, and 300 mOsmol (Fig. 2). For recordings of synaptic potentials, the intracellular solution contained the following (in mm): 120 KMeSO_4_, 10 KCl, 0.5 EGTA, 10 HEPES, 2.5 MgATP, 0.3 NaGTP, 10 Na2-phosphocreatine, 0.1 spermine, and 0.1% biocytin, pH 7.3–7.4, and 280 ± 5 mOsmol (Fig. 5). Data was acquired using a Multiclamp 700B amplifier (Molecular Devices) and digitized using Digidata 1440A and pClamp 10 (Molecular Devices). Recordings were low-pass filtered at 2 kHz and digitized at 20 kHz. Series resistance was regularly monitored during experiments and data were included only if the holding current and series resistance were stable.

Membrane properties of EYFP-labeled SOM-INs were measured in current-clamp recordings (Tricoire et al., 2011). Resting membrane potential was measured with the holding current *I* = 0 pA immediately after break-in in whole-cell configuration. Input resistance (*R*m) was measured using a linear regression of voltage deflections (±15 mV) in response to current steps of 5 pA increment (holding membrane potential −60 mV). Membrane time constant was calculated from the mean responses to 20 successive hyperpolarizing current pulses (20 pA; 400 ms) and was determined by fitting voltage responses with a single exponential function. Action potential (AP) threshold was taken as the voltage at which the slope trajectory reached 10 mV/ms, whereas AP amplitude was the difference in membrane potential between threshold and peak; these properties were measured for the first AP elicited by a depolarizing 800-ms-long current pulse just sufficient to bring the cell to threshold for AP generation. Firing frequency was calculated from the AP number during an AP train elicited by an 800-ms-long current injection at twice threshold. The sag index was determined from a series of negative current steps (800 ms duration). From the *V*–*I* plots, the peak negative voltage deflection (*V*hyp) and the steady-state voltage deflection (*V*sag, calculated for the last 200 ms of the current step) were used to calculate the index as the ratio *V*rest − *V*sag/*V*rest − *V*hyp, for current injections corresponding to *V*sag = −80 mV.

EPSCs were evoked in interneurons using constant current pulses (50 μs duration) via an ACSF-filled bipolar theta-glass electrode positioned approximately 100 μm lateral to the recorded cell soma. EPSCs were recorded in the presence of (2R)-amino-5-phosphonovaleric acid (AP5; 50 μM) and gabazine (5 μM) to block NMDA and GABA_A_ receptors, respectively. Putative single-fiber EPSCs were evoked at 0.5 Hz using minimal stimulation (failure rate >50%). LTP was induced by three episodes (given at 30 s intervals) of TBS of afferent fibers (5 bursts each consisting of 4 pulses at 100 Hz with 250 ms interburst interval) paired with postsynaptic depolarization (5 depolarizing steps to −20 mV, 60 ms long). EPSPs were evoked at 0.1 Hz using constant current pulses (50 μs duration) through a concentric bipolar Pt/Ir electrode (FHC) positioned in the stratum oriens close to the alveus, approximately 100 μm lateral to the recorded cell soma. Membrane potential was held at −60 mV by constant current injection. EPSPs were evoked during a hyperpolarizing current step (5–10 mV, 0.5–1 s duration) to avoid action potential generation. LTP was induced by the same TBS protocol described above for voltage-clamp experiments, except that it was not paired with any postsynaptic depolarization or hyperpolarization. EPSPs were recorded in ACSF in the absence of the antagonists AP5 and gabazine. In some experiments, LY367385 (40 μM; Tocris Bioscience) was added to the extracellular solution. EPSCs and EPSPs were usually characterized in one cell per slice, and the different experimental conditions were interleaved. Responses were analyzed off-line using Clampfit (pClamp 10; Molecular Devices), GraphPad, and OriginPro 8. Amplitude of EPSC and EPSP (average response including failures), failure rate (failures/total stimulations), and potency (amplitude excluding failures) of EPSCs, were averaged in 5 min bins over the total 35 min period of recordings.

### Field potential recordings and optogenetics

To express archaerhodopsin-3/GFP (ArChR3/GFP) in somatostatin interneurons, heterozygous SOM-IRES-Cre;ArChR3/GFP mice were obtained by crossing SOM-IRES-Cre and ArChR3/GFP Ai35 mice. For experiments with field potential recordings, transverse hippocampal slices (400 μm thickness) were prepared from SOM-IRES-Cre;ArChR3/GFP mice as described above, except ice-cold oxygenated ACSF containing 1.3 mM MgSO_4_ and 2.5 mM CaCl_2_. The slices were allowed to recover for at least 2 h at 32°C in ACSF, and for an additional 30 minutes at 27°–28°C while submerged in a recording chamber continuously perfused (2–2.5 ml/min) with ACSF. Field EPSPs (fEPSPs) were recorded in CA1 stratum radiatum with glass electrodes (1–2.5 MΩ) filled with ASCF. Schaffer collaterals were stimulated (0.1 ms duration; 30 sec^−1^) using a concentric bipolar tungsten stimulating electrode (FHC) placed in stratum radiatum proximal to the CA3 region. A second concentric bipolar tungsten stimulating electrode was positioned in the oriens–alveus junction proximal to the subiculum for theta-burst conditioning trains (as described above). Field potentials were recorded with a differential extracellular amplifier (Microelectrode AC Amplifier Model 1800, A-M Systems), filtered at 2 kHz, digitized at 10 kHz (Digidata 1440A), and analyzed with pClamp10 (Molecular Devices). Stimulus intensity was adjusted to elicit 50% of maximal fEPSP. fEPSP slope was measured at 10–90% of fEPSP amplitude.

LTP was induced at CA1 Schaffer collateral synapses by high-frequency stimulation (HFS) using a train (1s duration) of 100 Hz pulses. A conditioning TBS consisting of three episodes (given at 30 s intervals) of five bursts (each consisting of four pulses at 100 Hz with 250 ms interburst interval) was applied at the oriens/alveus border to induce plasticity in SOM-INs in stratum oriens. ArChR3 was activated by illumination using a light guide positioned above the slice (590 nm yellow light, custom-made LED system). The measured LED power was 26 mW at the end of a 1 mm light guide. Data are expressed as mean ± SEM.

## Results

### Distribution of YFP-labeled interneurons and specific colocalization with SOM and PV

**Figure 1.**
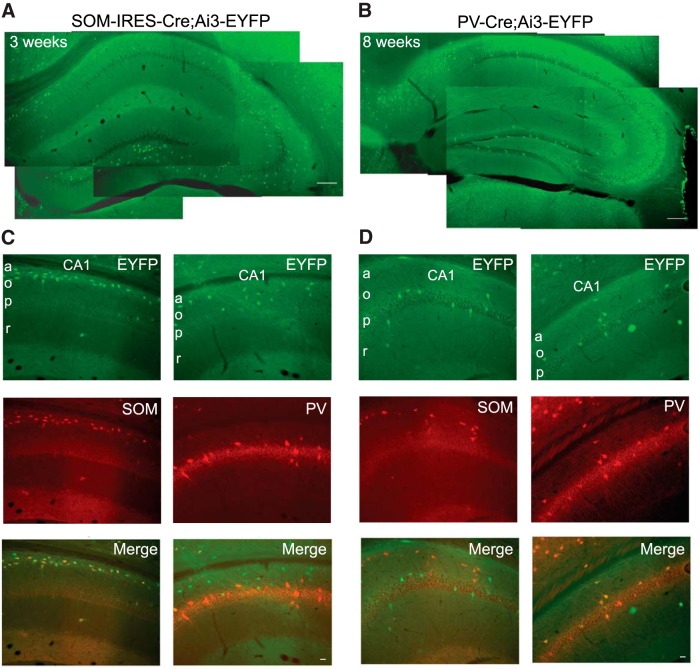
**Distribution of EYFP-labeled CA1 interneurons and specific colocalization with SOM and PV. *A***, ***B***, Montage of fluorescence images showing the mostly nonoverlapping distribution of EYFP-labeled INs in the hippocampus from SOM-IRES-Cre;Ai3-EYFP (***A***) and PV-Cre;Ai3-EYFP (***B***) mice. Scale bars, 100 µm. In the CA1 region, EYFP-labeled INs of SOM-IRES-Cre;Ai3-EYFP mice are present mostly in the oriens and alveus regions, whereas EYFP-labeled INs of PV-Cre;Ai3-EYFP mice are found near or in the pyramidal cell layer. ***C***, ***D***, Representative examples of specific colocalization of EYFP-labeled INs (top, green) from SOM-IRES-Cre;Ai3-EYFP (***C***) and PV-Cre;Ai3-EYFP (***D***) mice with immunofluorescence for somatostatin (middle left, red) and parvalbumin (middle right, red), respectively. Merged images are shown at bottom. Scale bars, 10 µm. Nearly all CA1 EYFP-labeled INs from SOM-IRES-Cre;Ai3-EYFP mice colocalized with somatostatin but not parvalbumin (***C***). Conversely, mostly all CA1 EYFP-labeled INs from PV-Cre;Ai3-EYFP mice were immunopositive for parvalbumin but not somatostatin (***D***).

SOM-INs or PV-INs were specifically labeled by breeding Ai3-EYFP reporter mice with SOM-IRES-Cre or PV-Cre mice, respectively. The distribution of EYFP-labelled SOM-INs and PV-INs interneurons was examined by fluorescence microscopy and their colocalization with somatostatin or parvalbumin was determined by immunofluorescence. Consistent with previous work (for review, see Freund and Buzsáki, 1996), the distribution of SOM-INs and PV-INs in the CA1 hippocampus was mostly nonoverlapping. EYFP-labeled SOM-IN somas were located mostly in stratum oriens and alveus of the CA1 and CA3 regions, as well as in the hilus of the dentate gyrus (Fig. 1*A*). EYFP-labeled PV-IN somas were mainly found in and around the pyramidal cell layer of CA1 and CA3 regions, and the granule cell layer of the dentate gyrus (Fig. 1*B*). We next verified the cell-specificity of the EYFP-labeling of CA1 interneurons by immunofluorescence. In SOM-IRES-Cre;Ai3-EYFP mice (Fig. 1*C*), 98.5% of EYFP-labeled interneurons in the CA1 region were immunopositive for somatostatin (*n* = 323 cells, 3 animals from 2 different litters), whereas only 6.9% of them were positive for parvalbumin (*n* = 354). In contrast in PV-Cre;Ai3-EYFP mice (Fig. 1*D*), 97.8% of EYFP-labeled CA1 interneurons were immunopositive for parvalbumin (*n* = 267 cells, 3 animals from 2 different litters), and only 10.7% were positive for somatostatin (*n* = 193 cells), thus confirming the specific labeling of mostly nonoverlapping CA1 populations of dendrite-projecting SOM-INs and perisomatic projecting PV-INs (Freund and Buzsáki, 1996; Tricoire et al., 2011) in the mice lines.

### LTP at the excitatory synapses onto SOM-INs

**Figure 2.**
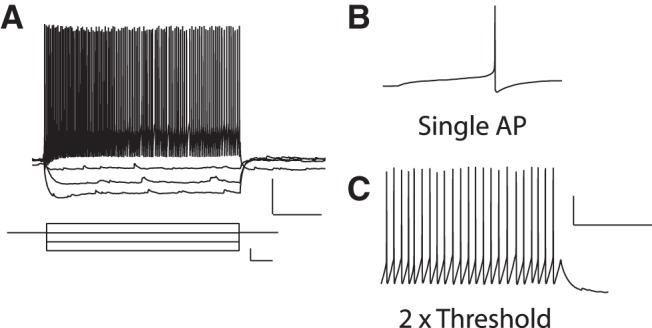
**Membrane properties of CA1 EYFP-labeled SOM-INs. *A*,** Example of voltage responses (top; scale bars: 20 mV, 200 ms) during current-clamp recordings evoked by current steps (bottom; scale bars: 40 pA, 100 ms) of varying amplitude from a representative CA1 EYFP-labeled SOM-IN (held at Vm of −60 mV). ***B***, ***C*,** Representative examples of traces with single action potential (***B***) and repetitive firing (at 2× threshold; ***C***) evoked by current-pulse injections. Scale bars: 20 mV, 200 ms.

Next we made use of SOM-IRES-Cre;Ai3-EYFP mice to determine whether the Hebbian LTP described at synapses onto CA1 oriens-alveus interneurons (Perez et al., 2001) occurs at excitatory synapses onto SOM-INs. First we characterized with whole-cell current-clamp recordings the membrane properties of CA1 EYFP-labeled SOM-INs (Fig. 2). The resting membrane potential was −58.3 ± 1.9 mV (*n* = 8), the membrane resistance was 194.8 ± 20.6 MΩ (*n* = 9), the membrane time constant was 24.6 ± 2.8 ms (*n* = 10), the action potential half-width and amplitude were 0.44 ± 0.05 ms (*n* = 13) and 66.5 ± 2.3 mV (*n* = 13), respectively, the action potential threshold was −40.4 ± 1.7 mV (*n* = 13) and the average firing frequency in response to depolarizing pulses was 23.6 ± 2.0 Hz at 2× threshold (*n* = 13). The cells also displayed a membrane sag at hyperpolarized membrane potentials (0.92 ± 0.01 sag index; *n* = 9). These properties are consistent with those previously reported for SOM-INs identified by mRNA expression (Tricoire et al., 2011).

**Figure 3.**
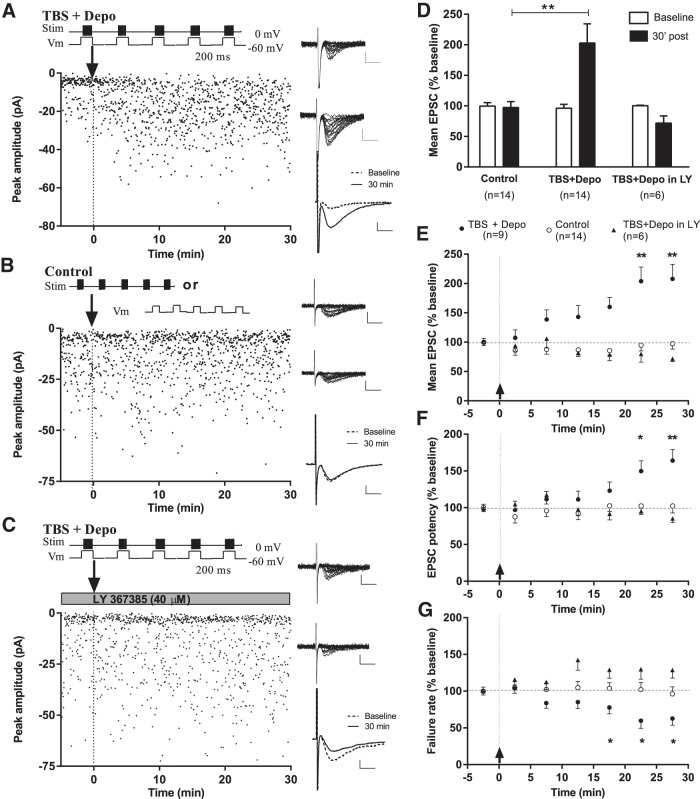
**mGluR1a-dependent Hebbian LTP at excitatory synapses onto CA1 EYFP-labeled SOM-INs. *A*–*C***, Diagrams (top, left) showing the stimulation pairing protocol for LTP induction (***A***; theta-burst stimulation paired with postsynaptic depolarization; TBS + Depo), the control stimulation protocols (***B***; TBS or postsynaptic depolarization alone) and the stimulation pairing protocol in the presence of 40 μm LY367385, an mGluR1a antagonist (***C***; TBS + Depo in LY). EPSC amplitude time plots (bottom, left) from representative CA1 EYFP-labeled SOM-INs showing increase in EPSC amplitude after the pairing protocol (***A***) but not after control stimulation (***B***; TBS alone in this particular example) nor in the presence of LY367385 (***C***). Twenty consecutive EPSC traces from respective cells from baseline period (top, right) and 30 min poststimulation (middle, right). Scale bars: 20 pA, 5 ms. Superimposed average traces (of 100 individual events, including failures; bottom, right; scale bars: 5 pA, 5 ms) illustrating the increase in response after the pairing protocol (***A***) but not control stimulation (***B***) nor in the presence of LY367385 (***C***). ***D***, Summary bar graph for all cells, showing no change in EPSC amplitude (including failures) after control stimulation, increase 30 min after the pairing protocol, and no change after the pairing protocol in the presence of LY367385. ANOVA, **p = 0.0025. ***E*–*G***, Summary time plots of EPSC measures (5 min bins) showing gradual development of LTP over time in all cells showing LTP after pairing (*n* = 9), but not in cells with control stimulation (*n* = 14) nor in cells with the pairing stimulation in the presence of LY367385 (*n* = 6). LTP was manifested as an increase in EPSC amplitude (including failures; ***E***) and potency (***F***), and a decrease in failure rate (***G***). ANOVA and Dunnett’s multiple-comparison tests; *p < 0.05, **p < 0.01.

Then we used whole-cell voltage-clamp recordings of EPSCs evoked by minimal stimulation of putative single-fibers to examine if excitatory synapses onto CA1 EYFP-labeled SOM-INs show Hebbian LTP. Pairing of theta-burst stimulation with postsynaptic depolarization (TBS + Depo) produced an increase in EPSC amplitude (average EPSC including failures) to 202.9 ± 31.3% of baseline at 30 min postinduction (paired *t* test, *p* = 0.0025^a^, *n* = 14; Fig. 3*A*,*D*). Control stimulation, consisting of theta-burst stimulation (*n* = 7) or depolarization (*n* = 7) alone, did not produce lasting changes in EPSC amplitude (96.0 ± 11.1% of baseline at 30 min postinduction for pooled controls; paired *t* test, *p* = 0.75^b^, *n* = 14; Fig. 3*B*,*D*).

When examined in individual cells, LTP occurred in 9 of 14 cells that received the pairing stimulation protocol (TBS + Depo), so the development of LTP over time was analyzed in these cells (Fig. 3*E*–*G*). A post-test for linear trend revealed that EPSC amplitude (including failures), potency (EPSC amplitude excluding failures) and failure rate changed gradually over the 5–30 min period after LTP induction (ANOVA, *p* < 0.0001^c^, *n* = 9). EPSC amplitude was increased at 20–25 min (203.7 ± 24.2% of baseline) and 25–30 min [207.7 ± 25.9% of baseline; repeated-measures (rm) ANOVA, *p* = 0.0016^d^, and Dunnett’s multiple-comparison test, *n* = 9; Fig. 3*E*]. EPSC potency was increased at 20–25 min (149.7 ± 14.1% of baseline) and 25–30 min (164.0 ± 15.3% of baseline; rmANOVA, *p* = 0.0025^e^, and Dunnett’s multiple-comparison test, *n* = 9; Fig. 3*F*). EPSC failure rate was decreased at 15–20 min (77.8 ± 8.5% of baseline), 20–25 min (59.8 ± 10.4% of baseline), and 25–30 min (62.7 ± 9.1% of baseline; rmANOVA, *p* = 0.011^f^, and Dunnett’s multiple-comparison test, *n* = 9; Fig. 3*G*). In cells with control stimulation, there were no significant changes over time in EPSC amplitude (96.0 ± 11.1% of baseline at 30 min; rmANOVA, *p* = 0.7512^g^; *n* = 14), potency (100.9 ± 7.5% of baseline at 30 min, rmANOVA, *p* = 0.9456^h^) or failure rate (97.1 ± 6.3% of baseline at 30 min, rmANOVA, *p* = 0.6528^i^; Fig. 3*E*–*G*).

These results show that CA1 SOM-IN excitatory synapses show a gradual late-onset LTP expressed as an increase in EPSC amplitude and potency, as well as a decrease in EPSC failure rate after the Hebbian pairing protocol.

### LTP at SOM-IN excitatory synapses depends on mGluR1a

SOM-INs in CA1 specifically express mGluR1a at high level (Baude et al., 1993; Somogyi and Klausberger, 2005) and Hebbian LTP in oriens-alveus interneurons is dependent on mGluR1a activation (Perez et al., 2001). Therefore, we examined whether LTP in CA1 EYFP-labeled SOM-INs was also mGluR1a dependent, using the mGluR1a antagonist LY 367385. Application of the pairing protocol, TBS, and depolarization, in the presence of LY 367385 (40 μM) failed to produce LTP (Fig. 3*C*,*D*). At 30 min postinduction, there were no significant changes in EPSC amplitude (78.8 ± 4.9% of baseline; rmANOVA, *p* = 0.0846^j^, *n* = 6; Fig. 3*C*–*E*), EPSC potency (85.2 ± 5.7% of baseline; rmANOVA, *p* = 0.3121^k^, *n* = 6; Fig. 3*F*), and failure rate (128.7 ± 11.0% of baseline; rmANOVA, *p* = 0.1212^l^, *n* = 6; Fig. 3*G*).

### Absence of LTP at synapses onto PV-INs

**Figure 4.**
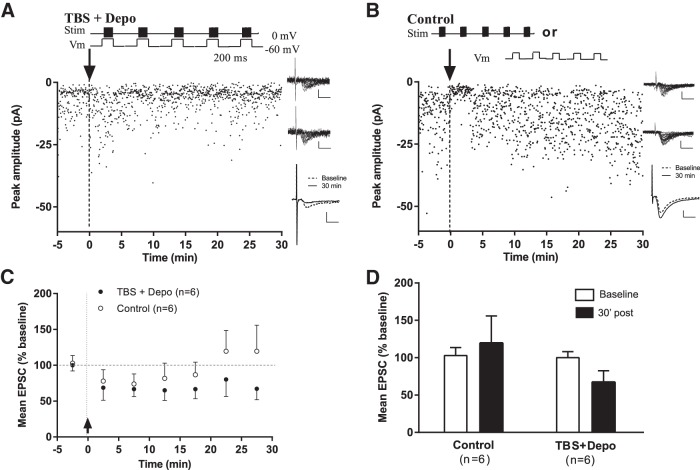
**Absence of LTP at the excitatory synapses onto CA1 EYFP-labeled PV-INs. *A***, ***B***, Diagrams (top) showing the pairing protocol for LTP induction (***A***; theta-burst stimulation paired with postsynaptic depolarization; TBS + Depo) and the control stimulation protocols (***B***; TBS or postsynaptic depolarization alone). EPSC amplitude time plots (bottom, left) from representative CA1 EYFP-labeled PV-INs showing no increase in EPSC amplitude after the pairing protocol (***A***) nor after control stimulation (***B***; TBS alone in this example). Twenty individual traces from respective cells during baseline period (top, right) and 30 min poststimulation (middle, right). Scale bars: 10 pA, 5 ms. Superimposed average traces (of 100 individual events, including failures; bottom, right; scale bars: 5 pA, 5 ms) illustrating the failure to increase responses after the pairing protocol (***A***) or control stimulation (***B***). ***C***, Summary time plots of EPSC amplitude (5 min bins) for all cells showing no change over time in EPSC amplitude (including failures) after the pairing protocol (*n* = 6) or control stimulation (*n* = 6). ANOVA and Dunnett’s multiple-comparison tests, *p* > 0.05. ***D***, Summary bar graphs for all cells showing lack of LTP in PV-INs after the pairing protocol (*n* = 6; ANOVA, *p* = 0.205) or control stimulation (*n* = 6; *p* = 0.633).

PV-INs are another distinct subpopulation of interneurons with perisomatic projections to pyramidal cells (Freund and Buzsáki, 1996). Next we used a similar approach but with whole-cell recordings from CA1 EYFP-labeled PV-INs obtained from PV-Cre;Ai3-EYFP mice to determine whether Hebbian mGluR1a-mediated LTP was also present in this interneuron type or whether it was cell-type-specific. Pairing of theta-burst stimulation with postsynaptic depolarization (TBS + Depo; Fig. 4*A*,*C*) failed to produce gradual changes in EPSC amplitude in CA1 EYFP-labeled PV-INs over 30 min postinduction (average EPSC including failures: 67.8 ± 15.4% of baseline at 30 min postinduction; paired *t* test, *p* = 0.2057^m^; *n* = 6; Fig. 4*D*). Similarly, control stimulation, consisting of theta-burst stimulation (*n* = 4) or depolarization (*n* = 2) alone (Fig. 4*B*,*C*), did not produce lasting changes in EPSC amplitude (119.0 ± 33.6% of baseline at 30 min postinduction for pooled controls; paired *t* test, *p* = 0.6333^n^; *n* = 6; Fig. 4*D*). These results reveal that Hebbian mGluR1a-mediated LTP is absent from afferent inputs to another large population of CA1 interneurons, the PV-INs, and thus shows cell-type specificity for SOM-INs synapses.

### Facilitation of CA1 Schaffer collateral pathway LTP by TBS in oriens-alveus

**Figure 5.**
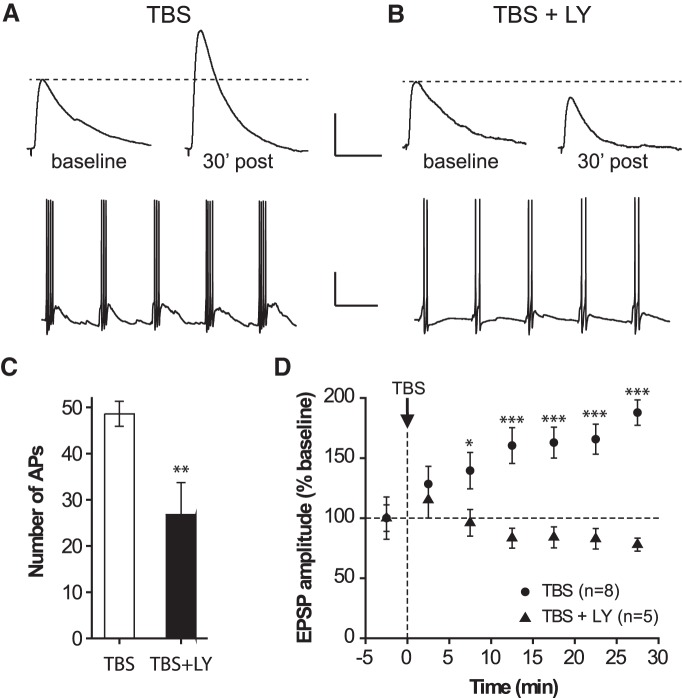
**mGluR1a-dependent LTP in current-clamp recordings with intact glutamatergic and GABAergic transmission. *A*, *B***, Averaged EPSPs from 30 consecutive responses during the baseline period (top, left) and 30 min after TBS (top, right) in the absence (***A***) and presence (***B***) of LY367385 from representative EYFP-labeled SOM-INs. Firing patterns during TBS (bottom). Note the prolonged depolarization underlying each burst in control conditions (***A***), which is abolished in the presence of LY367385 (***B***). Scale bars: top, 2 mV, 50 ms; bottom, 20 mV, 200 ms. ***C***, Bar graph showing the decreased number of APs elicited during the TBS protocol in the absence (*n* = 8) and presence (*n* = 5) of LY367385 (Student’s test). ***D***, Summary time plot of EPSP amplitude (5 min bins) for all cells, showing the gradual development of LTP over time, which is blocked in the presence of LY367385 (rmANOVA, *p* = 0.001, and Dunnett’s multiple-comparison tests from baseline). *p < 0.05, **p < 0.01, ***p < 0.001.

Next we investigated the potential role of Hebbian LTP at SOM-IN synapses in the function of the CA1 local circuitry. Activation of dendrite-targeting OLM interneurons, a major subgroup of SOM-INs, differentially regulates LTP at major inputs to CA1 pyramidal cells, reducing LTP in the temporo-ammonic pathway from entorhinal cortex and facilitating LTP in the Schaffer collateral pathway from CA3 (Leão et al., 2012). It has been previously shown that TBS in oriens-alveus, via Hebbian LTP at interneuron input synapses, resulted in long-term enhancement of evoked firing in oriens-alveus interneurons during cell-attached recordings (Croce et al., 2010), as well as long-term enhancement of polysynaptic inhibition of pyramidal cells (Lapointe et al., 2004). Therefore, we tested if TBS in oriens-alveus could produce mGluR1a-mediated Hebbian LTP at SOM-IN synapses and result in a long-term regulation of LTP in CA1 Schaffer collateral pathway using field recordings. First we established, using whole-cell current clamp recordings from SOM-IRES-Cre;Ai3-EYFP mice, that TBS in oriens-alveus induces LTP of excitatory synapses onto SOM-IN in the same conditions used for field recordings (bulk stimulating electrode, absence of postsynaptic depolarization, absence of glutamatergic and GABAergic antagonists, see Materials and Methods). In these conditions, TBS in oriens-alveus produced a gradual increase (linear regression, ANOVA, *p* < 0.001^°^, *n* = 8) in EPSP amplitude (average EPSP including failures) reaching 187.8 ± 10.6% of baseline at 30 min postinduction (rmANOVA, *p* < 0.001^p^, and Dunnett’s multiple-comparison tests, *n* = 8; Fig. 5*A*,*D*). Bath application of 40 µM LY367385 decreased the number of action potentials elicited during the TBS protocol (without LY, 48.6 ± 2.7 action potentials, *n* = 8; with LY, 26.8 ± 6.9 action potentials, *n* = 5; Student’s test, *p* = 0.005^q^; Fig. 5*A*–*C*) and prevented TBS-induced LTP of EPSP amplitude. After TBS in oriens-alveus in the presence of LY367385, EPSP amplitude was 78 ± 5.4% of baseline at 30 min postinduction (rmANOVA, *p* = 0.0007^r^, and Dunnett’s multiple-comparison tests, *n* = 5; Fig. 5*B*,*D*). These results demonstrate that mGluR1a-mediated LTP at excitatory synapses onto SOM-INs occurs in conditions for field potential recordings of LTP in the Schaffer collateral pathway.

**Figure 6.**
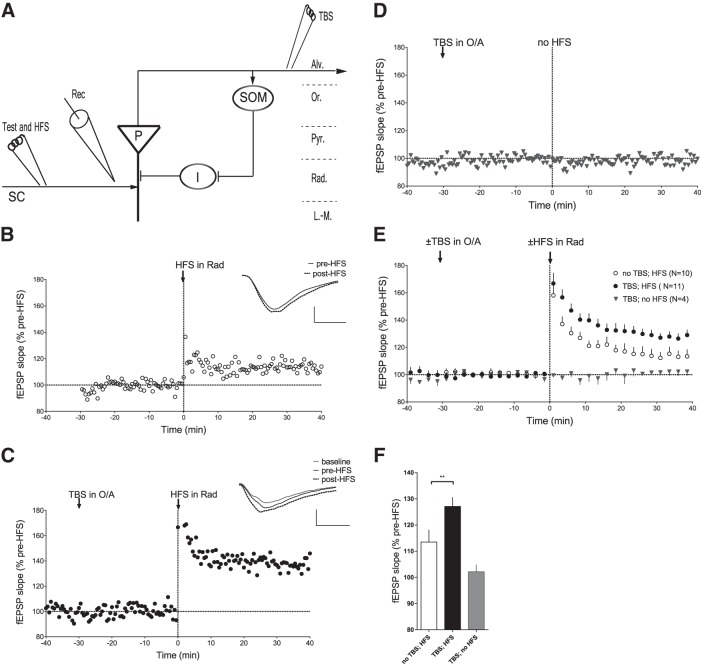
**Regulation of Schaffer collateral pathway LTP by theta-burst stimulation in oriens-alveus. *A***. Diagram of experimental arrangement of extracellular recording and stimulation electrodes, and targeted CA1 pathways and cells. P, Pyramidal cell; I, inhibitory interneuron; SC, Schaffer collateral pathway; Rec, recording electrode; Alv, alveus; Or, stratum oriens; Pyr, stratum pyramidale; Rad, stratum radiatum; L-M, stratum lacunosum/moleculare. Arrowheads and bars refer to excitatory and inhibitory synapses, respectively. **B–D**, Time plots of Schaffer collateral fEPSP slope from individual representative slices from SOM-IRES-Cre;ArChR3/GFP mice showing LTP induced by HFS in stratum radiatum (***B***), enhanced LTP when HFS induction was preceded by a conditioning TBS in stratum oriens-alveus 30 min earlier (***C***), and no effect of TBS alone (***D***). Insets (***B***, ***C***) are average fEPSPs (of 30 individual traces) during baseline, pre-HFS, and 30 min post-HFS. Scale bars: 0.5 mV, 5 ms. ***E***, Summary fEPSP slope time plots for all slices, showing larger magnitude of HFS-induced LTP when preceded by TBS in stratum oriens-alveus. ***F***, Summary bar graph showing increased HFS-induced LTP of fEPSP slope at 30 min postinduction after a conditioning TBS in oriens-alveus (TBS; HFS) relative to control without TBS (no TBS; HFS, ANOVA, **p = 0.0052), and no effects of TBS on fEPSPs in experiments without HFS.

We then investigated whether LTP at SOM-INs synapses could produce a long-term regulation of LTP in the Schaffer collateral pathway using field recordings. In addition, for these experiments we used SOM-IRES-Cre;ArChR3/GFP mice for cell-specific expression in SOM-INs of archaerhodopsin-3 (ArCh3), an outward proton pump that causes hyperpolarization, so we could manipulate selectively SOM-IN excitability using optogenetics during field recording experiments. First, low-frequency Schaffer collateral stimulation was given in stratum radiatum to elicit CA1 fEPSPs during a baseline period (30 min; Fig. 6*A*,*B*). HFS (100 Hz, 1 s) of Schaffer collaterals was then applied, resulting in LTP of fEPSP slope (113.5 ± 4.7% of baseline at 30 min postinduction; *n* = 10, paired *t* test, *p* = 0.010^s^; Fig. 6*B*,*E*,*F*). In comparison, application of TBS in oriens-alveus resulted in enhancement of Schaffer collateral pathway LTP tested 30 min later (Fig. 6*A*,*C*). TBS in oriens-alveus did not affect Schaffer collateral evoked fEPSPs during the baseline period, indicating no effect on basal transmission (Fig. 6*D*). However, LTP induction in the Schaffer collateral pathway given 30 min after TBS in oriens-alveus, resulted in an increase in fEPSP slope (128.2 ± 3.5% of baseline, *n* = 11; paired *t* test, *p* = 0.0001^t^; Fig. 6*C*,*E*) that was greater than in the control condition without TBS (ANOVA, *p* = 0.0052^u^; Fig. 6*F*). The effect of TBS in oriens-alveus was tested also on Schaffer collateral pathway for the same time period but without subsequent LTP induction. TBS in oriens-alveus had no effect on Schaffer collateral fEPSPs recorded for a similar duration (102.2 ± 2.6% of baseline; *n* = 4; paired *t* test, *p* = 0.5794^v^; Fig. 6*D*–*F*). These results indicate that TBS in oriens-alveus does not affect basal transmission at CA3–CA1 synapses, but has a long-lasting effect to enhance LTP in the Schaffer collateral pathway.

### TBS-induced enhancement of Schaffer collateral LTP is mediated by SOM-INs

The observed TBS-induced enhancement of Schaffer collateral pathway LTP is consistent with TBS inducing mGluR1a-mediated Hebbian LTP at SOM-INs synapses, and resulting in increased SOM-IN facilitation of LTP in the Schaffer collateral pathway from CA3 (Leão et al., 2012).

**Figure 7.**
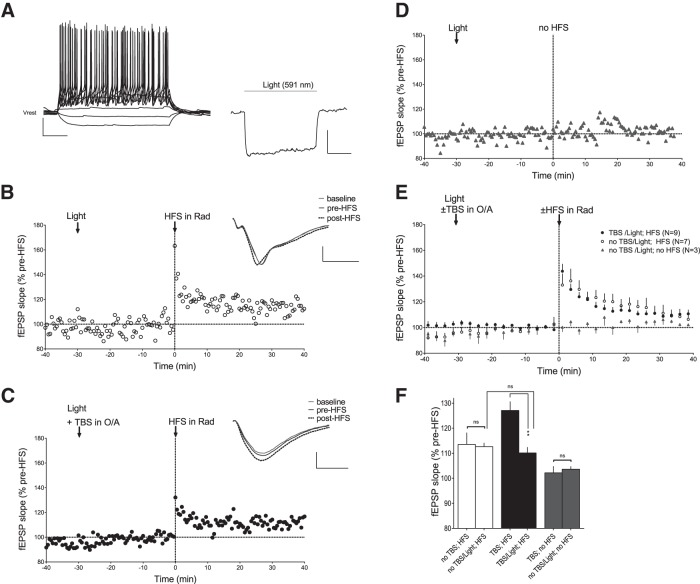
**TBS-induced enhancement of Schaffer collateral pathway LTP is prevented by SOM-INs hyperpolarization. *A***, Whole-cell current-clamp recordings from a representative GFP-expressing SOM-IN in acute slice of SOM-IRES-Cre;ArChR3/GFP mouse, showing responses to current step injections (left) and membrane hyperpolarization in response to wide-field yellow light (591 nm) exposition (right). Scale bars: left, 20 mV, 100 ms; right, 10 mV, 500 ms. ***B*–*D***, Time plots of Schaffer collateral fEPSP slope from representative slices from SOM-IRES-Cre;ArChR3/GFP mice showing similar LTP induced by HFS in stratum radiatum when preceded by yellow light exposition 30 min earlier (***B***) and by conditioning TBS in stratum oriens-alveus during yellow light exposition (***C***), and no effect of yellow light exposition alone on fEPSPs (***D***). Insets (***B***, ***C***) are average fEPSPs (of 30 individual traces) from baseline, pre-HFS, and 30 min post-HFS. Scale bars: 0.5 mV, 5 ms. ***E***, Summary fEPSP slope time plots for all slices, showing no enhancement of HFS-induced LTP when TBS in stratum oriens-alveus is given during yellow light exposition. ***F***, Summary bar graph showing similar HFS-induced LTP of fEPSP slope at 30 min postinduction after a conditioning TBS in oriens-alveus with versus without yellow light exposition (ANOVA, *p* = 0.0751; n.s.), and no effects of light alone on fEPSPs in experiments without HFS.

We next investigated whether the TBS-induced enhancement of CA1 Schaffer collateral pathway LTP was dependent on SOM-INs in SOM-IRES-Cre;ArChR3/GFP mice using optogenetics to selectively hyperpolarize SOM-INs during TBS. In control whole-cell recordings from GFP-expressing SOM-INs in slices from SOM-IRES-Cre;ArChR3/GFP mice, yellow light (591 nm wavelength) illumination of the slice through an optic fiber induced membrane hyperpolarization for the duration of light stimulation (Fig. 7*A*; 1.5 s, *n* = 5 cells).

Next we examined the TBS-induced modulation of Schaffer collateral pathway LTP in the presence of yellow light stimulation. First as control, we verified that prior application of light alone (1.5 min duration) did not affect Schaffer collateral pathway LTP (Fig. 7*B*). In these conditions, illumination did not affect fEPSPs during the baseline period and HFS caused an increase in fEPSPs slope (112.7 ± 1.5% of baseline at 30 min postinduction; *n* = 9; paired *t* test, *p* = 0.0001^w^; Fig. 7*E*,*F*) similar to previous results testing HFS alone (Fig. 7*F*). Interestingly, TBS in oriens-alveus given during light stimulation did not affect fEPSPs during the baseline period, but failed to enhance HFS-induced LTP (Fig. 7*C*). HFS given 30 min after TBS in oriens-alveus during light stimulation, resulted in an increase in fEPSP slope (110.1 ± 2.3% of baseline at 30 min postinduction; *n* = 7; paired *t* test, *p* = 0.0030^x^; Fig. 7*E*,*F*) that was not different from that without TBS (ANOVA, *p* = 0.0751^y^; Fig. 7*F*). Light stimulation alone had no effect on Schaffer collateral fEPSPs recorded for a similar period (103.6 ± 1.1% of baseline; paired *t* test, p=0.0690^z^; *n* = 3; Fig. 7*D*–*F*). These results indicate that TBS-induced enhancement of Schaffer collateral pathway LTP is prevented when TBS is given during light-activated hyperpolarization of SOM-INs, suggesting that SOM-INs activation during TBS is required for TBS-induced enhancement of Schaffer collateral LTP.

### TBS-induced enhancement of Schaffer collateral LTP is mGluR1a-dependent

The impairment of TBS-induced enhancement of Schaffer collateral pathway LTP by light inactivation of SOM-INs during TBS provides further support that TBS may be inducing mGluR1a-mediated Hebbian LTP at SOM-INs synapses to result in increased SOM-IN facilitation of LTP in the Schaffer collateral pathway from CA3. Therefore we next investigated if TBS-induced enhancement of CA1 Schaffer collateral pathway LTP required activation of mGluR1a, using the mGluR1a antagonist LY367385, which blocks LTP in SOM-INs (Figs. 3, 5).

**Figure 8.**
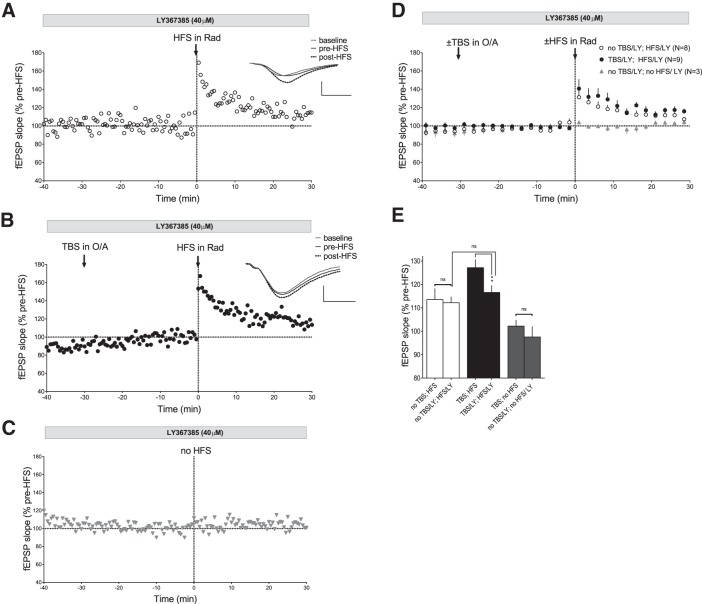
**TBS-induced enhancement of Schaffer collateral pathway LTP is prevented by mGluR1a blockade. *A*–*C***, Time plots of Schaffer collateral fEPSP slope from representative slices from SOM-IRES-Cre;ArChR3/GFP mice showing that, in the presence of the mGluR1a antagonist LY367385, LTP induced by HFS in stratum radiatum (***A***) is similar to LTP induced by HFS which is preceded by conditioning TBS in stratum oriens-alveus 30 min earlier (***B***), and that fEPSPs are unaffected during long-term recordings in the presence of LY367385 (***C***). Insets (***A***, ***B***) are average fEPSPs (of 30 individual traces) from baseline, pre-HFS, and 30 min post-HFS. Scale bars: 0.5 mV, 5 ms. ***D*.** Summary fEPSP slope time plots for all slices, showing no enhancement of HFS-induced LTP when TBS in stratum oriens-alveus is given in the presence of LY367385. ***E***, Summary bar graph showing similar HFS-induced LTP of fEPSP slope at 30 min postinduction whether preceded or not by a conditioning TBS in oriens-alveus in the presence of LY367385 (ANOVA followed by Dunnett’s multiple-comparison test, *p* > 0.05), lack of TBS-induced enhancement of Schaffer collateral pathway LTP in the presence of LY367385 (ANOVA, **p = 0.0052) and no effects of LY367385 alone on fEPSPs in experiments without HFS.

Application of LY367385 did not affect Schaffer collateral pathway LTP (Fig. 8*A*). In the presence of LY367385, HFS caused an increase in fEPSPs slope (112.2 ± 4.5% of baseline at 30 min postinduction; *n* = 8; paired *t* test, *p* = 0.0050^aa^; Fig. 8*D*,*E*) similar to previously found with HFS alone (Fig. 8*E*). Importantly, TBS in oriens-alveus in the presence of the mGluR1a antagonist LY367385 failed to enhance HFS-induced LTP (Fig. 8*B*). HFS given 30 min after TBS in LY367385 resulted in an increase in fEPSP slope (116.0 ± 3.0% of baseline at 30 min postinduction; *n* = 9; paired *t* test, *p* = 0.0005^ab^; Fig. 8*D*,*E*) that was not different from that without TBS (ANOVA followed by Dunnett’s multiple-comparison test, *p* > 0.05; Fig. 8*E*). Application of LY367385 alone had no effect on Schaffer collateral fEPSPs recorded for a similar period (97.6 ± 9.0% of baseline; paired *t* test, *p* = 0.4856^ac^; *n* = 4; Fig. 8*C*–*E*). These results indicate that mGluR1a activation during TBS is required for TBS-induced enhancement of Schaffer collateral LTP.

Collectively, these series of experiments show that TBS-induced enhancement of Schaffer collateral pathway LTP is prevented by light inactivation of SOM-INs and by mGluR1a antagonism (Figs, 7, 8), suggesting TBS in oriens-alveus produces mGluR1a-mediated Hebbian LTP at SOM-INs synapses and results in a long-term upregulation of LTP in CA1 Schaffer collateral pathway (Table 1).

**Table 1.** Statistical table

	Data structure	Type of test	Power
a	Normal distribution	paired *t* test	0.0025
b	Normal distribution	paired *t* test	0.75
c	Normal distribution	ANOVA	0.0001
d	Normal distribution	rmANOVA, Dunnett's *post hoc*	0.0016
e	Normal distribution	rmANOVA, Dunnett's *post hoc*	0.0025
f	Normal distribution	rmANOVA, Dunnett's *post hoc*	0.011
g	Normal distribution	rmANOVA	0.7512
h	Normal distribution	rmANOVA	0.9456
i	Normal distribution	rmANOVA	0.6528
j	Normal distribution	rmANOVA	0.0846
k	Normal distribution	rmANOVA	0.3121
l	Normal distribution	rmANOVA	0.1212
m	Normal distribution	paired *t* test	0.2057
n	Normal distribution	paired *t* test	0.6333
o	Normal distribution	ANOVA	0.0005
p	Normal distribution	rmANOVA, Dunnett’s *post hoc*	2.66E-7
q	Normal distribution	*t* test	0.0053
r	Normal distribution	rmANOVA, Dunnett’s *post hoc*	0.0007
s	Normal distribution	paired *t* test	0.01
t	Normal distribution	paired *t* test	0.0001
u	Normal distribution	paired *t* test	0.0052
v	Normal distribution	paired *t* test	0.5794
w	Normal distribution	paired *t* test	0.0001
x	Normal distribution	paired *t* test	0.003
y	Normal distribution	rmANOVA, Dunnett's *post hoc*	0.0751
z	Normal distribution	paired *t* test	0.069
aa	Normal distribution	paired *t* test	0.005
ab	Normal distribution	paired *t* test	0.0005
ac	Normal distribution	paired *t* test	0.4856

## Discussion

In the present study, we have demonstrated a Hebbian form of LTP occurring at excitatory synapses onto identified SOM-INs in the hippocampal CA1 area. This form of LTP was dependent on mGluR1a and did not occur in PV-INs. In addition, our results revealed that prior induction of mGluR1a-dependent LTP in SOM-INs enhanced the magnitude of LTP at SC synapses in pyramidal cells. These results uncover a novel metaplastic function of O/A SOM-INs conferring to them the ability to modulate durably CA1 network activity and plasticity.

### mGluR1a Hebbian LTP at excitatory feedback synapses onto SOM-INs

The large diversity of interneurons within the cerebral cortex has been a major hurdle for the detailed investigation of the function of synaptic plasticity in various interneurons. The emergence and development of Cre-lox system for recombination of reporter genes has made possible to target selectively interneuron classes by taking advantage of their specific pattern of protein expression. This approach allowed us to visualize and target two major, mainly nonoverlapping, classes of hippocampal CA1 interneurons: (1) the dendrite-targeting SOM-INs of the O/A region, corresponding largely to the OLM and bistratified cells; and (2) the perisomatic projecting PV-INs located within and nearby the pyramidal layer, corresponding mainly to the basket and axoaxonic cells (for review, see Freund and Buzsáki, 1996; Klausberger and Somogyi, 2008; Tricoire et al., 2011). We demonstrated that TBS episodes delivered to the O/A region, to stimulate CA1 pyramidal cell axon collateral excitatory inputs, induce LTP in O/A SOM-INs but not in PV-INs. This result extends the interneuron subtype specificity of LTP that has been previously described in the heterogeneous interneuron population of the O/A region (Perez et al., 2001; Lapointe et al., 2004; Croce et al., 2010).

As previously demonstrated, this type of LTP was induced in O/A SOM-INs by pairing presynaptic theta-burst stimulation in the O/A region and postsynaptic depolarization, and was dependent on mGluR1a (Perez et al., 2001; Lapointe et al., 2004). Independent on NMDARs, this form of LTP has been called mGluR1a Hebbian LTP, in contrast to the canonical Hebbian NMDAR-dependent LTP (Ouardouz and Lacaille, 1995; Lamsa et al., 2005) and to the anti-Hebbian CP-AMPAR-dependent LTP requiring postsynaptic hyperpolarization (Lamsa et al., 2007). These three distinct types of LTP occur at excitatory feedback synapses from CA1 pyramidal cells onto interneurons in the O/A region (for review, see Pelletier and Lacaille, 2008; Kullmann et al., 2012; Topolnik, 2012). PV-INs also receive such feedback excitation and it has been reported recently that these synapses display both canonical Hebbian and anti-Hebbian LTP (Le Roux et al., 2013). However, we show here that brief theta-burst stimulation episodes failed to induce Hebbian LTP at excitatory feedback synapses onto PV-INs, probably because this class of interneurons mainly expresses mGluR5 rather than mGluR1 (van Hooft et al., 2000). Our findings thus reveal that theta-burst stimulation induces cell-type-specific mGluR1a-mediated Hebbian plasticity at the excitatory inputs onto SOM-INs, but not PV-INs.

We observed mGluR1a Hebbian LTP in approximately 65% of the synaptic connections tested in SOM-INs with minimal stimulation and voltage clamp recordings, consistently with previous findings (Lapointe et al., 2004). This may be due to the fact that, in addition to the predominant feedback pathway, the O/A region also contains fibers from CA2/3 pyramidal cells (Ishizuka et al., 1990; Blasco-Ibáñez and Freund, 1995; Wittner et al., 2007) providing feedforward excitation to O/A-INs through CI-AMPARs and these synapses do not display mGluR1a Hebbian LTP (Croce et al., 2010), indicating a dual regulation of synaptic inputs in these interneurons. Nonetheless, it has been previously shown that TBS delivered in the O/A region reliably induces postsynaptic firing of O/A interneurons in cell-attached recordings and results in a mGluR1a-mediated long-lasting potentiation of synaptically evoked firing of O/A cells (Croce et al., 2010). The present findings thus indicate that mGluR1a Hebbian LTP of feedback excitatory inputs to SOM-INs translates into a durable increase in their output firing, which made it a key component in the regulation of the input–output function in SOM-INs.

### Metaplasticity in the CA1 hippocampal network

Understanding the specific contributions of the various GABAergic interneuron subtypes in the control of information flow within the cortico-hippocampal network is an important question. SOM-INs constitute a group of interneurons sharing the property to inhibit pyramidal cell and interneuron dendrites conferring them a crucial role in regulating the gain of pyramidal neuron input–output transformations (Pouille and Scanziani, 2004). Experiments using pharmacogenetic and optogenetic approaches have demonstrated that a specific silencing of hippocampal CA1 SOM-INs increases pyramidal cells firing rates and burst spiking during SC stimulation *in vitro* (Lovett-Barron et al., 2012; but see Wilson et al., 2012 for an opposite role in the visual cortex) and during spatial mapping *in vivo* (Royer et al., 2012).

However, SOM-INs are a nonhomogeneous population. For example, in hippocampal CA1, OLM cells densely synapse within the SLM to inhibit the pyramidal cell distal dendritic tuft, at the level of their temporo-ammonic inputs from the entorhinal cortex, whereas bistratified cells inhibit more proximal pyramidal cell dendrites in the stratum radiatum where they receive their inputs from SC (for review, see Klausberger, 2009; Müller and Remy, 2014). It has been shown that OLM neurons, themselves being less homogeneous than initially thought (Chittajallu et al., 2013), restrict voltage signals by providing a direct inhibition to the pyramidal cell dendritic tuft in response to temporo-ammonic stimulation, preventing their propagation along proximal dendrites (Leão et al., 2012). In addition, specific optogenetic stimulation of OLM cells increase SC-evoked excitatory synaptic responses and LTP by disinhibiting these synapses in pyramidal cells (Leão et al., 2012). Indeed, OLM cells also inhibit several classes of interneuron (Katona et al., 1999; Elfant et al., 2008; for review, see Chamberland and Topolnik, 2012), including SC-associated interneurons at the border of the stratum radiatum and the SLM, and to a lesser extent, bistratified cells (Leão et al., 2012), that are both coaligned with SC inputs. The present results reveal a form of metaplasticity in which mGluR1a LTP occurring at excitatory synapses onto SOM-INs specifically increases LTP magnitude at SC-PC synapses. This suggests that mGluR1a-mediated LTP induction in SOM-INs provide a disinhibition of SC–PC synapses, likely via the inhibition of interneurons in the stratum radiatum, and support an interneuron input-specific control of pyramidal cell integrative function. In our field potential recording experiments, TBS in oriens may have induced NMDAR-mediated LTP at synapses onto PV-INs (Le Roux et al., 2013). However, because selective optogenetic inactivation of SOM-INs during TBS or antagonism of mGluR1a receptors prevented the metaplasticity of LTP in the SC pathway, our results suggest that non-mGluR1a-mediated LTP at synapses onto PV-INs may not contribute to this metaplasticity.

Other forms of disinhibitory metaplasticity have been reported in CA1 PCs, but with a direct disinhibition of SC-pyramidal cells synapses through LTD of inhibitory synapses (I-LTD), i.e. plasticity of the output synapses of interneurons. They involve a specific contribution of cholecystokinin-expressing interneurons of the stratum radiatum in the dynamic segregation of SC and temporo-ammonic inputs (Basu et al., 2013), with a prominent role of group I mGluRs (Chevaleyre and Castillo, 2003, 2004; Basu et al., 2013; Xu et al., 2014), associated with the ability to support temporal associative memories (Xu et al., 2014). Taking into consideration that a learning episode increases intrinsic excitability in CA1 SOM-INs (McKay et al., 2013), this suggests that these interneurons may be endowed with multiple plasticity mechanisms to increase output function (input synapses, intrinsic excitability, and output synapses), probably acting in concerted fashion.

### Consequences for learning and memory

A growing body of information is accumulating about the specific role of SOM-INs in cognitive abilities, mainly occurring via disinhibition mechanisms. In the anterior cingulate cortex, they participate in decision making (Kvitsiani et al., 2013) and they support and modulate sensory processing in different neocortical areas through a VIP-SOM-IN-dependent disinhibition of principal cells (Lee et al., 2013; Pi et al., 2013; Zhang et al., 2014). They mediate precise visual processing by inhibiting PV-INs in the primary visual cortex (Cottam et al., 2013). They are inhibited by PV-INs to disinhibit PC dendrites in the basolateral amygdala during auditory-cued fear conditioning (Wolff et al., 2014); they mediate fear learning and produce fear expression through disinhibition mechanisms in the central amygdala (Li et al., 2013; Penzo et al., 2015). Within the hippocampus, CA1 OLM and bistratified cells rhythmically modulate pyramidal neurons activity *in vivo* (Klausberger et al., 2003, 2004; Katona et al., 2014). During fear conditioning, SOM-INs from the CA1 area, in particular the OLM cells, are believed to respond specifically to the unconditioned aversive stimulus, and by a direct inhibition, restrict the size of the coding pyramidal cell population (Lovett-Barron et al., 2014). Similarly, contextual fear conditioning increases the number of mossy fibers synaptic contacts onto interneurons in CA3, restricting the size of the coding pyramidal population and promoting memory precision (Ruediger et al., 2011). In the present study, we show that LTP occurring at excitatory synapses onto SOM-INs increases the magnitude of subsequent LTP at SC-PC synapses. This suggests that a disinhibition might occur in a subpopulation of principal neurons displaying an increased LTP (see above in Discussion). However, it does not exclude that repetitive pyramidal firing could also durably increase feedback and lateral direct inhibition to a different subpopulation of pyramidal neurons (Dupret et al., 2013).

Finally, a late form of LTP, induced by mGluR1a activation, dependent on transcription and translation via the mTORC1 pathway and lasting at least 24 h, has recently been shown in CA1 O/A INs (Ran et al., 2009, 2012), suggesting that a persistent form of LTP may play a crucial role in the long-lasting metaplastic regulation of CA1 network activity during hippocampus-dependent learning and memory.

## Synthesis

The decision was a result of the Reviewing Editor Marlene Bartos and the peer reviewers coming together and discussing their recommendations until a consensus was reached. A fact-based synthesis statement explaining their decision and outlining what is needed to prepare a revision is listed below. The following reviewer(s) agreed to reveal their identity: Melanie Woodin

Synthesis of Reviews:

Both reviewers praised the study on metaplasticity on CA1 interneurons in particular the identification of the interneuron type showing this form of plastic changes. However both reviewers raised some major and minor comments which the authors should carefully into account in their revised manuscript. In particular it was questioned whether the block of NMDARs may be a possible mechanisms underlying the loss of LTP at synaptic inputs onto PV-cells. Moreover, the conditions of LTP induction in somatostain-expressing interneurons were questioned. Please view the main points below:

Reviewer 1:

The present study demonstrates that theta-burst stimulation induces a Hebbian form of LTP in SOM-INs; moreover, they demonstrate that this plasticity depends on mGluR1a activation. Using optogenetics they show that this interneuron plasticity facilitates LTP in the Schaffer collateral pathway of pyramidal cells. Taken together this study elucidates metaplasticity in the CA1 microcircuit. Overall, this is study makes an important contribution to the literature and is well executed. I only have minor concerns.

- In the Results section under the subheading "LTP at the excitatory synapses onto SOM-INs" the authors report membrane properties identified INs. However, the refer to these properties as "low (RMP)", "high (membrane resistance)", "fast (membrane time constant" etc. What are "low, high, and fast" in comparison to? Pyramidal neuron properties? Other IN properties? The authors should either remove these terms (low, high, fast, etc), or indicate what they are in comparison to.

- In the Results text reporting p-values there are superscripts after several p values, what are these?

- In the Results text describing Figure 3 the authors state "potency (excluding failures)" this should be rephrased to "potency (amplitude excluding failures).

- The last sentence of the first paragraph on p15 starting with "For these experiments, we used SOM-IRES-Cre..." I recommend adding a sentence that explains the rational for using this strategy.

Reviewer 2:

This paper studies the consequences of LTP at excitatory inputs onto CA1 oriens/alveus interneurons. They report that the previously described mGluR1-dependent LTP onto CA1 oriens/alveus interneurons occurs onto interneurons expressing somatostatin but not onto parvalbumin-expressing interneurons. They then show that the LTP onto somatostatin interneurons does not affect the strength of Schaffer collateral inputs onto CA1 pyramidal neurons, but facilitates subsequent LTP induction at this synapse.

The experiments are well designed and performed and the results are generally convincing. However, I have several points that should be addressed in order to strengthen the main conclusions of the study:

The authors show that the mGluR1-dependent LTP onto oriens/alveus interneurons occurs onto somatostatin interneurons. They show that induction of this plasticity mediates a metaplastic facilitation of SC-CA1 LTP. While the facilitation is predictable based on a previous model suggesting that OLM interneuron activation facilitate SC LTP, the experiments on LTP onto SOM interneurons and on LTP at SC-CA1 synapses are performed in very different conditions.

1- In particular, the authors claim that a TBS failed to induce LTP at excitatory synapses onto PV interneurons. However, these experiments are performed in presence of NMDA antagonist. NMDA-dependent LTP onto PV interneurons has been reported and it is unclear whether any LTP would be induced onto PV cells under the conditions used in this study without NMDA blockers.

2- Similarly, it is unclear whether LTP onto SOM interneurons occurs in the conditions used to study LTP at SC-CA1 inputs. The authors should show that in the conditions used for SC LTP (no blockers for GABA and NMDA receptors, bipolar stimulating electrode instead of glass pipette), LTP indeed occurs onto SOM interneurons.

3- The authors propose that the increase in firing of SOM interneurons after LTP provides a dis-inhibition of SC-CA1 synapses, via inhibition of interneurons in stratum radiatum. This model has been proposed for OLM cells (Leao et al, 2012). However, we do not know whether the mGluR1-LTP occurs onto SOM OLM cells or onto other SOM interneurons (i.e, bistratified cells). Increased activity in OLM interneurons has been shown to increase the LTP of SC-CA1 inputs, but also to decrease LTP at distal cortical inputs onto CA1. It would be nice to show whether LTP of distal inputs is also affected following mGluR1-LTP onto SOM interneurons.

We thank the Editors and the Reviewers for judging the manuscript potentially suitable for publication and for their positive and constructive comments to improve the manuscript. Suggestions have been taken into account in the revised manuscript (eN-NWR-0051-15). Please find below a point-by-point reply to the reviewers' concerns.

Manuscript Instructions

- The sex of the species studied is not mentioned in the materials and methods section. Please mention "male", "female", "x males and x females", "of either sex".

A sentence indicating that experiments were performed in mice of either sex was added (p 6 para 1).

Reviewer 1:

We thank reviewer 1 for considering that "Overall, this is study makes an important contribution to the literature and is well executed." We addressed the other points raised by the reviewer in the following manner:

1- In the Results section under the subheading "LTP at the excitatory synapses onto SOM-INs" the authors report membrane properties identified INs. However, they refer to these properties as "low (RMP)", "high (membrane resistance)", "fast (membrane time constant" etc. What are "low, high, and fast" in comparison to? Pyramidal neuron properties? Other IN properties? The authors should either remove these terms (low, high, fast, etc), or indicate what they are in comparison to.

As suggested by the reviewer, the comparative terms "low, high and fast" have been removed from the text for clarity (p 13 para 1).

2- In the Results text reporting p-values there are superscripts after several p values, what are these?

In the Results text, superscripts refer to the statistical table provided at the end of the Result section, in compliance with eNeuro information for Authors, p. 8, Results paragraph: "Authors must provide (...) specific p values (...) followed by a superscript lowercase letter referring to the statistical table provided at the end of the results section."

3- In the Results text describing Figure 3 the authors state "potency (excluding failures)" this should be rephrased to "potency (amplitude excluding failures).

As correctly pointed out by the reviewer, we rephrased as suggested (p. 14, para 1).

4- The last sentence of the first paragraph on p15 starting with "For these experiments, we used SOM-IRES-Cre..." I recommend adding a sentence that explains the rational for using this strategy.

As recommended by the reviewer, we added clarifications in two sentences explaining the rationale for using the strategy of optogenetics and field recordings in the Results section (p. 17, last para) and (p. 18, last para).

Reviewer 2:

We thank the reviewer 2 for considering that "The experiments are well designed and performed and the results are generally convincing." We addressed the other concerns of the reviewer as follows:

1- In particular, the authors claim that a TBS failed to induce LTP at excitatory synapses onto PV interneurons. However, these experiments are performed in presence of NMDA antagonist. NMDA-dependent LTP onto PV interneurons has been reported and it is unclear whether any LTP would be induced onto PV cells under the conditions used in this study without NMDA blockers.

The reviewer was correct in pointing out confusion in the text about the intent of these experiments. We apologize for the confusion and corrected the text. We had concluded correctly (p 16, first para) that "These results reveal that Hebbian mGluR1a-mediated LTP is absent from afferent inputs to another large population of CA1 interneurons, the PV-INs..." (see also, p 5 last para Introduction; p 23 first para Discussion). So the intent of these experiments was to investigate the specific contribution of mGluR1a-mediated LTP in SOM-INs in the regulation of the CA1 network. In order to achieve this, we first established that this particular form of LTP is cell-type specific and occurs in SOM-INs but not in PV-INs. We corrected the introductory statement for these specific experiments (p 15, beginning of last para) to indicate that we examined if Hebbian mGluR1a-mediated LTP was also present in PV-INs.

It is important to stress that we do not claim that PV-INs are unable to display LTP. To the contrary, it is well known that excitatory synapses onto PV-INs can perform both Hebbian NMDAR-mediated and anti-Hebbian CP-AMPAR-mediated LTP, depending on their feedforward or feedback inputs (Nissen et al., 2010; LeRoux et al., 2013). In the present study, we demonstrated that PV-INs synapses do not display mGluR1a-mediated TBS-induced LTP in the absence of functional NMDARs. Moreover, the main finding of the present study resides in the specific metaplastic control of the CA1 Schaffer collateral pathway by mGluR1a-mediated plasticity in SOM-INs. In this context, it is important to highlight that even if TBS in oriens triggers some type of LTP in PV-INs, it does not affect LTP in the Schaffer collateral pathway because the TBS-induced metaplasticity we observed is completely blocked by light-mediated hyperpolarization of SOM-INs and is mGluR1a-mediated, excluding a complementary contribution of other types of non-mGluR1a-mediated plasticity in PV-INs in this process. We added sentences in the Discussion section (p. 26, end para 1) to clarify this point.

2- Similarly, it is unclear whether LTP onto SOM interneurons occurs in the conditions used to study LTP at SC-CA1 inputs. The authors should show that in the conditions used for SC LTP (no blockers for GABA and NMDA receptors, bipolar stimulating electrode instead of glass pipette), LTP indeed occurs onto SOM interneurons.

The reviewer was correct in pointing out these important experiments. As suggested, we carried out these additional experiments. We assessed whether TBS in oriens is able to induce LTP in SOM-INs using a concentric bipolar electrode, in the absence of NMDARs and GABAARs antagonists. We show using current clamp recordings that LTP indeed occurs at synapses onto SOM-INs following TBS episodes in oriens in these specific experimental conditions. This LTP showed a similar slow onset time profile than LTP recorded in voltage-clamp with minimally-evoked EPSCs under NMDAR and GABAAR antagonists. Also in these additional current-clamp experiments, we show that TBS in oriens fails to induce LTP in the presence of the mGluR1a antagonist LY367385 (40 ÂµM) establishing it is mGluR1a-mediated (see p. 16 last para and p. 17 first para for results, new Figure 5). Together these additional experiments indicate that Hebbian mGluR1a-mediated LTP occurs at synapses onto SOM-INs after TBS in our field experiments and likely underlies the observed increase in LTP magnitude in the Schaffer collateral pathway. Methods (p 8-10), Results (p 16-17), Statistic Table, Legends and Figures (new Fig 5; and modified numbering figs 6-8 throughout the manuscript) were modified to include these new results.

3- The authors propose that the increase in firing of SOM interneurons after LTP provides a dis-inhibition of SC-CA1 synapses, via inhibition of interneurons in stratum radiatum. This model has been proposed for OLM cells (Leao et al, 2012). However, we do not know whether the mGluR1-LTP occurs onto SOM OLM cells or onto other SOM interneurons (i.e, bistratified cells). Increased activity in OLM interneurons has been shown to increase the LTP of SC-CA1 inputs, but also to decrease LTP at distal cortical inputs onto CA1. It would be nice to show whether LTP of distal inputs is also affected following mGluR1-LTP onto SOM interneurons.

We agree with the reviewer that these additional experiments would be interesting to carry out. The functional dissociation between SOM-expressing OLM and bistratified cells is an important and interesting question that can be measured at many levels and this issue has been addressed in the discussion section of the present work. In addition the work of Leao and colleagues (2011) showing a bidirectional modulation of CA3 and entorhinal inputs after specific activation of OLM cells was also discussed. In the present study, we did not intend to specifically investigate any functional dissociation between SOM-IN subtypes and we did not perform a systematic characterization of SOM-IN subtype identity regarding their ability to display mGluR1a-mediated LTP. We consider that further experiments to functionally dissociate distinct SOM-expressing interneuron subtypes, together with the bidirectional regulation of pyramidal excitatory inputs, are not essential to support the main conclusions of our study and are thus beyond the scope of the present paper.
